# Prognostic risk signature based on the expression of three m6A RNA methylation regulatory genes in kidney renal papillary cell carcinoma

**DOI:** 10.18632/aging.104053

**Published:** 2020-11-07

**Authors:** Zhuolun Sun, Changying Jing, Chutian Xiao, Tengcheng Li, Yu Wang

**Affiliations:** 1 Department of Urology, Third Affiliated Hospital, Sun Yat-Sen University, Guangzhou 510630, China; 2 Eye Institute of Shandong University of Traditional Chinese Medicine, Jinan 250002, China

**Keywords:** m6A, RNA methylation, kidney renal papillary cell carcinoma, survival analysis, prognostic signature

## Abstract

In this study, we investigated the prognostic significance of the expression of N6-methyladenosine (m6A) RNA methylation regulatory genes in kidney renal papillary cell carcinoma (KIRP). RNA-sequencing data analysis showed that 14 of 20 major m6A RNA methylation regulatory genes were differentially expressed in the KIRP tissues from The Cancer Genome Atlas (TCGA) database. We constructed a prognostic risk signature with three m6A RNA methylation regulatory genes, *IGF2BP3, KIAA1429* and *HNRNPC*, based on the results from univariate and LASSO Cox regression analyses. Multivariate Cox regression analysis confirmed that the risk score based on the three-gene prognostic risk signature was an independent predictive factor in KIRP. The overall survival of high-risk KIRP patients was significantly shorter than the low-risk KIRP patients. Expression of the three prognostic risk-related genes correlated with the AJCC and TNM stages of KIRP patients from TCGA and GEPIA datasets. ROC curve analysis showed that the three-gene prognostic risk signature precisely predicted the 1-year, 3-year and 5-year survival of KIRP patients. These findings demonstrate that expression of three prognostic risk-related m6A RNA methylation regulatory genes accurately predicts survival outcomes in KIRP patients.

## INTRODUCTION

Renal cell carcinoma (RCC) accounts for approximately 90% of all kidney tumors and 2% to 3% of all adult malignancies [1]. Kidney renal papillary cell carcinoma (KIRP) is the second most frequent subtype of RCC and accounts for nearly 15% to 20% of the total RCC cases [2, 3]. KIRP is a heterogeneous disease with two histological subtypes that show significant variations in disease progression and survival outcomes [4, 5]. The underlying molecular mechanisms are not fully understood, despite the characterization of several gene mutations in KIRP tissues [6]. Furthermore, the efficacy of targeted therapy has not been established in KIRP patients with advanced disease [3]. Currently, there is no consensus on the optimal risk gene signature to determine the prognosis of KIRP patients [7]. Hence, there is an urgent need to identify novel prognostic biomarkers and therapeutic targets for improving the survival outcomes of KIRP patients.

DNA methylation and post-translational histone modifications are involved in the epigenetic regulation of cellular development and differentiation in normal and pathological conditions [8, 9]. Recent studies also show that RNA methylation epigenetically regulates several biological functions. [10, 11] The common RNA modifications are 5-methylcytosine (m5C), N6-methyladenosine (m6A), N7-methylguanosine (m7G), and pseudouridine [12–14]. The m6A RNA methylation is the most frequent, abundant, and conserved form of RNA methylation that has been reported in several messenger RNAs (mRNAs), long noncoding RNAs (lncRNAs) and other RNA species [12, 15]. Genome-wide changes in gene expression because of dynamic and reversible changes in m6A methylation have been reported in normal and disease conditions in various tissues [16]. Analogous to DNA methylation or histone modifications, m6A methylation is regulated by several methyltransferases, demethylases, and other RNA binding proteins [16]. Several methyltransferases (m6A writers) such as METTL3, METTL14, WTAP, KIAA1429, RBM15, RBM15B and ZC3H13 are involved in the generation of the m6A modification of mRNAs, lncRNAs and other RNAs [17]. On the other hand, m^6^A is removed by a demethylase (m6A eraser) composed of FTO and ALKBH5 [18]. The m^6^A modification alters the interactions of the modified RNAs with the RNA binding proteins (m6A readers), including IGF2BP1, IGF2BP2, IGF2BP3, YTHDF1, YTHDF2, YTHDF3, YTHDC1, YTHDC2, HNRNPC, HNRNPA2B1 and RBMX [15, 16, 18, 19]. The m6A RNA methylation regulatory proteins have been reported to play a critical role in stem cell differentiation and pluripotency, metabolism, circadian rhythm, embryo development, and tumor progression [13, 20, 21]. Aberrant m6A modification is associated with the progression of urological tumors [22].

The clinical relevance and prognostic significance of m6A-RNA methylation regulatory genes has not been studied in KIRP. Hence, we systematically analyzed the relationship between the expression of 20 different m6A RNA methylation regulatory genes and the clinicopathological parameters of KIRP patients from The Cancer Genome Atlas (TCGA) database. We also established a prognostic risk signature model with three m6A RNA methylation regulatory genes and evaluated its efficacy to predict survival outcomes of KIRP patients.

## RESULTS

### Fourteen out of twenty m6A RNA methylation regulatory genes are differentially expressed in KIRP tissues

**Figure 1:**
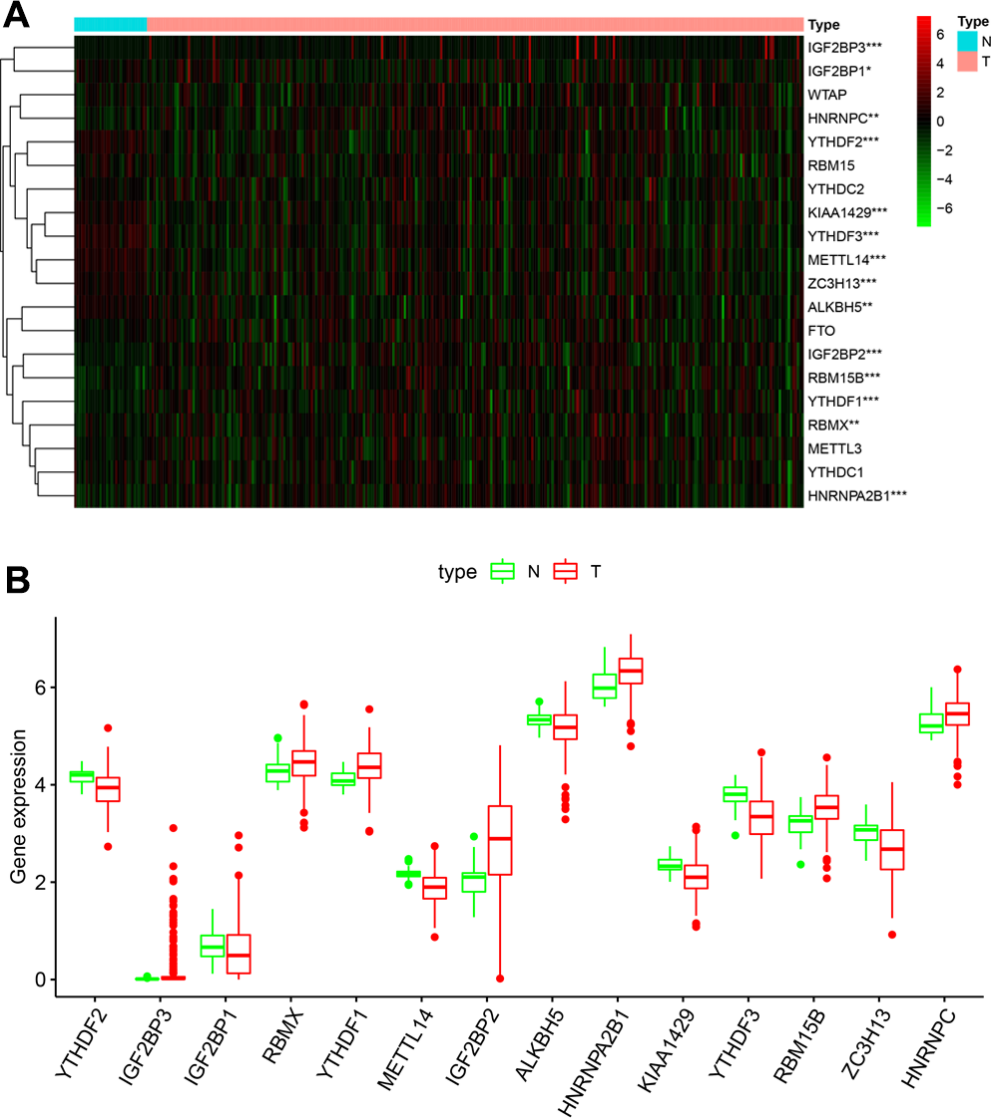
**Fourteen out of twenty m6A RNA methylation regulatory genes are differentially expressed in KIRP tissues.** (**A**) The heatmap demonstrates the expression of 20 m6A RNA methylation regulators in 289 KIRP and 32 normal kidney tissue samples from the TCGA database. The color bar from red to green denotes high to low gene expression. * P<0.05; ** P<0.01; *** P<0.001. (**B**) The boxplots show the expression of 14 differentially expressed m6A RNA methylation regulators in normal kidney and KIRP tissues from the TCGA database.

We analyzed the expression levels of 20 m6A RNA methylation regulating proteins in KIRP (n = 289) and normal samples (n = 32) from the TCGA database. The heatmap showed that the expression of 14 m6A methylation regulators (IGF2BP3, IGF2BP1, HNRNPC, YTHDF2, KIAA1429, YTHDF3, METTL14, ZC3H13, ALKBH5, IGF2BP2, RBM15B, YTHDF1, RBMX and HNRNPA2B1) were differentially expressed in KIRP tissues compared to normal kidney tissues (Figure 1A). We observed that IGF2BP3, RBMX, YTHDF1, IGF2BP2, HNRNPA2B1, RBM15B and HNRNPC were significantly upregulated, and YTHDF2, IGF2BP1, METTL14, ALKBH5, KIAA1429, YTHDF3 and ZC3H13 were significantly downregulated in the KIRP tissues compared to the normal kidney tissue samples (Figure 1B). The expression of WTAP, RBM15, YTHDC2, FTO, METTL3 and YTHDC1 was similar in KIRP and normal kidney tissue samples (Figure 1A).

### PPI network and correlation analysis between m6A RNA methylation regulators

**Figure 2:**
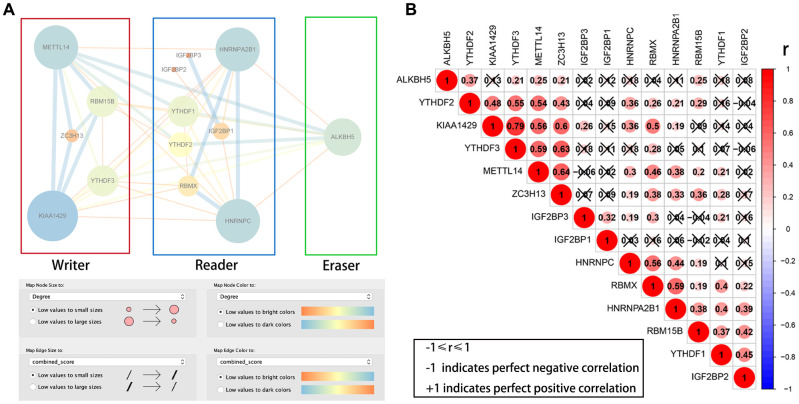
**PPI network and Pearson correlation analyses of 14 differentially expressed m6A RNA methylation regulatory genes.** (**A**) PPI network of the 14 differentially expressed m6A RNA methylation regulatory genes. (**B**) Pearson correlation analysis of the 14 differentially expressed m6A RNA methylation regulatory genes in the TCGA-KIRP cohort. Note: ‘r’ denotes Pearson correlation co-efficient whose value ranges between -1 (perfect negative correlation) and +1 (perfect positive correlation).

We then downloaded the data for the 14 differentially expressed m6A RNA methylation regulators from the STRING database and constructed a protein–protein interaction (PPI) network using Cytoscape. PPI network analysis showed that KIAA1429, HNRNPC, METTL14, HNRNPA2B1 and ALKBH5 were the hub genes (Figure 2A). The interaction between KIAA1429 and YTHDF3 (r = 0.79) was most significant among all the m6A RNA methylation regulators (Figure 2B).

### Identification of a prognostic risk signature based on three m6A RNA methylation regulators in the training cohort of KIRP patients

**Figure 3:**
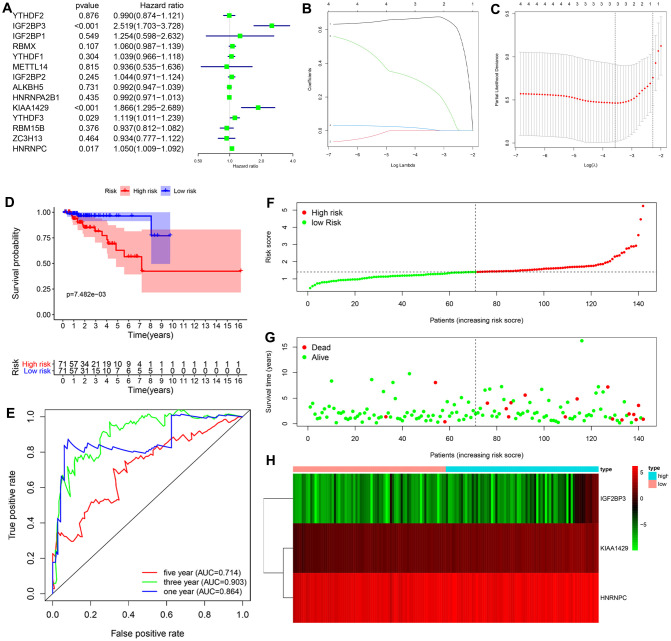
**Construction and evaluation of the 3-gene prognostic risk signature in the training cohort of KIRP patients.** (**A**) Univariate Cox regression analysis results show the p values and hazard ratios (HR) with confidence intervals (CI) of the 14 differentially expressed m6A RNA methylation regulatory genes. (**B**, **C**) LASSO Cox regression analysis results show the identification of the 3 prognostic risk signature genes. (**D**) Kaplan-Meier survival curves show the overall survival (OS) rates of high-risk (n=71) and low-risk (n=71) KIRP patients of the training cohort. The high-risk group shows shorter OS compared to the low-risk group. (**E**) ROC curve analysis results show the accuracy and reliability of the prognostic risk signature in determining the 1-year, 3-year, and 5-year survival outcomes of the high- and low-risk KIRP patients in the training cohort. The AUC values are shown in parenthesis. (**F**) The risk score distribution of the high-risk (red) and low-risk (green) KIRP patients in the training cohort. (**G**) The distributions of training cohort patients based on their survival times and risk scores. The red dots represent patients that have died, whereas, the green dots denote patients that are alive at the time of analysis. (**H**) The heatmap shows the expression levels of the three prognostic risk-related m6A RNA methylation regulators in the high-risk (blue) and low-risk (pink) KIRP patients of the training cohort.

Univariate Cox regression analysis of the transcriptome data from the TCGA-KIRP dataset showed that the expression of four m6A RNA methylation regulators (IGF2BP3, KIAA1429, YTHDF3 and HNRNPC) was significantly associated with the overall survival (OS) of KIRP patients (*P* < 0.05; Figure 3A). Furthermore, based on the results of the least absolute shrinkage and selection operator (LASSO) Cox regression analysis, we constructed a prognostic risk signature with three genes, including IGF2BP3, KIAA1429 and HNRNPC (Figure 3B, 3C).

Next, we analyzed the efficacy of the risk signature in predicting the prognosis of KIRP patients in the training cohort. We calculated the risk score for each patient in the training cohort and divided them into high-risk (n=71) and low-risk (n=71) groups according to the median risk score. Kaplan-Meier survival curve analysis showed that overall survival time was significantly shorter for KIRP patients in the high-risk group compared to the KIRP patients in the low-risk group (log-rank test *P* < 0.001; Figure 3D). The 3-yr OS rates for the high- and low-risk group were 81.3% and 96.3%, respectively. The 5-yr OS rates for the high- and low-risk group were 62.8%and 96.3%, respectively. The ROC curve analysis demonstrated significant prognostic predictive value for the risk signature in determining the 1-yr, 3-yr and 5-yr survival rates of KIRP patients (1-year AUC = 0.864, 3-year AUC = 0.903, 5-year AUC = 0.714; Figure 3E). The risk score distribution of the high- and low-risk group patients in the training cohort showed that the survival rates were significantly higher for the low-risk group compared to the high-risk group (Figure 3F, 3G). The heatmap showed higher expression levels of the three risk-related m6A RNA methylation regulators (IGF2BP3, KIAA1429 and HNRNPC) in the high- risk KIRP patients compared to the low-risk KIRP patient group (Figure 3H).

### Validation of the prognostic risk signature in the testing and entire TCGA-KIRP cohorts

We validated the accuracy and robustness of prognostic risk signature in the testing cohort and the entire TCGA-KIRP cohort. We calculated the prognostic risk scores of patients in both the testing (n=94) and the entire TCGA-KIRP (n=237) cohorts based on the prognostic risk signature and stratified the patients into high-risk (testing cohort: 47; entire TCGA cohort: 119) and low-risk (testing cohort: 47; entire TCGA cohort: 118) groups based on the median cut-off value. The detailed clinicopathological features of all the TCGA-KIRP patients are listed in Table 1.

**Table 1:** Characteristics of KIRP patients included in this study.

**Variable**	**Training cohort (n = 237)**	**Testing cohort (n = 94)**	**TCGA cohort (n = 331)**	***P***
	**Number (%)**	**Number (%)**	**Number (%)**	
**Age**				0.2569
≤65	93(61.58)	53(56.38)	146(61.34)	
>65	51(35.42)	41(43.62)	92(38.66)	
**Gender**				0.3097
Female	42(29.17)	21(22.34)	63(26.47)	
Male	102(70.83)	73(77.66)	175(73.53)	
**AJCC stage**				0.2672
I/II	113(78.47)	67(71.28)	180(75.63)	
III/IV	31(21.153)	27(28.72)	58(24.37)	
**T stage**				0.5685
T1-2	116(80.56)	72(76.60)	188(78.99)	
T3-4	28(21.53)	22(23.40)	50(21.01)	
**N stage**				0.2062
N0	125(86.81)	75(79.79)	200(84.03)	
N1-2	19(13.19)	19(20.21)	38(15.97)	
**M stage**				0.5844
M0	137(95.14)	87(92.55)	224(94.12)	
M1	7(4.86)	7(7.45)	14(5.88_	

**Figure 4:**
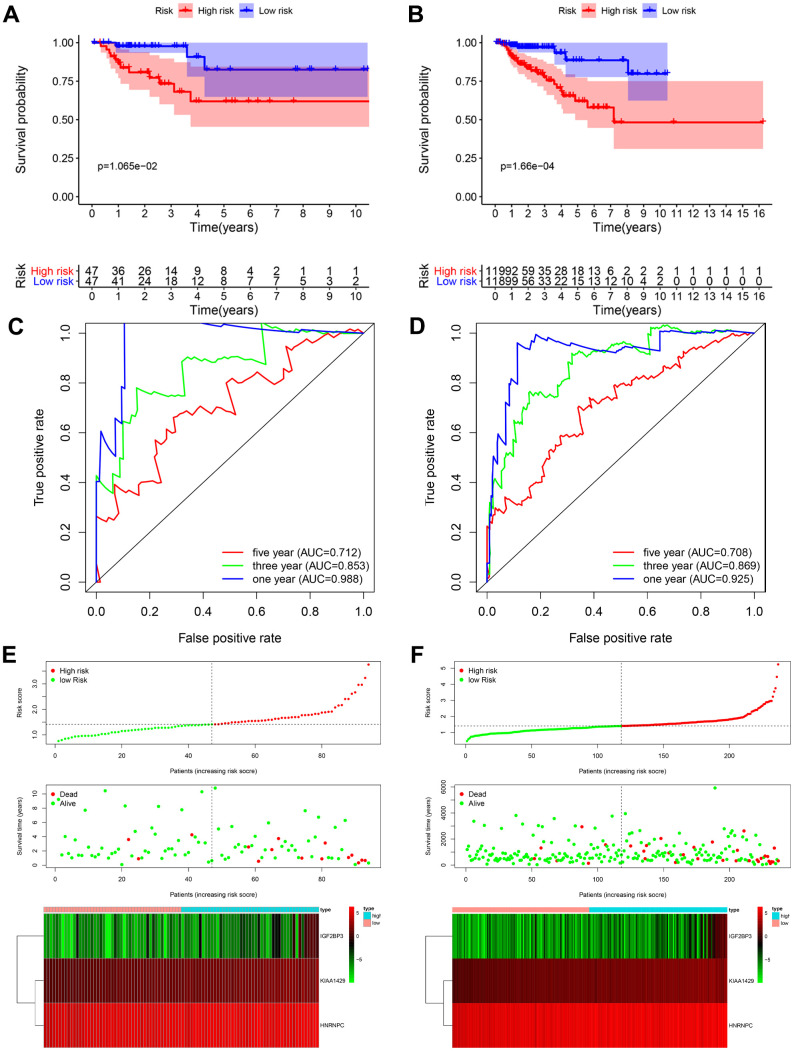
**Validation of the prognostic risk signature in the testing cohort and entire cohort.** (**A**) Kaplan-Meier curve analysis shows the overall survival rates of high-risk (n=47) and low-risk (n=47) KIRP patients in the testing cohort. (**B**) Kaplan-Meier curve analysis shows the overall survival rates of high-risk (n=119) and low-risk (n=118) KIRP patients in the entire TCGA cohort. (**C**, **D**) ROC curve analyses of the (**C**) testing cohort and (**D**) the entire TCGA-KIRP cohort show the false positive rate vs. true positive rate plots based on the prognostic risk signature. The AUC values for 1-year (blue), 3-year (green), and 5-year (red) survival rates are also shown. (**E**, **F**) The risk score distribution, survival status and prognostic risk gene expression in the (**E**) testing cohort and (**F**) entire TCGA-KIRP cohort is shown.

Kaplan-Meier survival curve analysis showed that the overall survival of high-risk patients were significantly shorter compared to the low-risk patients in both the testing cohort (*P* < 0.05; Figure 4A) and the entire TCGA-KIRP cohort (*P* < 0.001; Figure 4B). In the testing cohort, the 3-year and 5-year survival rates were shorter for the high-risk patients compared to the low-risk groups (3-year: 73.3% vs. 97.7%; 5-year: 61.9% vs. 82.5%). Similarly, in the entire TCGA-KIRP cohort, the 3-year and 5-year survival rates in high-risk group were lower than those in the low-risk group (3-year: 77.7 vs. 97.0%; 5-year: 62.0 vs. 88.6%). ROC curve analysis showed that the AUC values for the 1-, 3- and 5-year survival in the testing cohort were 0.988, 0.853 and 0.712, respectively (Figure 4C). The AUC values for the 1-, 3- and 5-year survival in the entire TCGA-KIRP cohort were 0.925, 0.869 and 0.708, respectively (Figure 4D). The risk score distribution, survival status and the risk gene expression in the testing cohort and the entire TCGA-KIRP cohort are shown in Figures 4E and 4F. Overall, our results showed that the prognostic risk signature accurately predicted the survival outcomes of KIRP patients.

### Independent prognostic value of the risk signature

To determine whether the risk signature can be used as an independent prognostic factor, Next, we performed univariate and multivariate Cox regression analyses to determine the independent prognostic significance of the risk score and the relevant clinicopathological factors, including age, gender, AJCC stage, T stage, N stage, and M stage. In the TCGA training cohort, univariate analyses showed that AJCC stage, T stage, N stage, M stage and risk score were significantly associated with OS. Subsequently, multivariate analyses showed that AJCC stage, T stage, M stage and risk score were significantly associated with OS (Table 2). Similar results were obtained for both the testing and the entire TCGA-KIRP cohorts (Table 2). These results demonstrate that the risk score calculated based on the prognostic risk signature is an independent prognostic factor in KIRP patients.

**Table 2:** Univariate and multivariate Cox regression analysis of clinical factors and prognostic risk signature for OS in the training, testing and entire cohort.

**Variable**	**Training cohort**				**Testing cohort**				**Entire cohort**			
	**Univariate**		**Multivariate**		**Univariate**		**Multivariate**		**Univariate**		**Multivariate**	
	**HR**	***P***	**HR**	***P***	**HR**	***P***	**HR**	***P***	**HR**	***P***	**HR**	***P***
**Age**												
≤65 vs >65	0.980	0.351	1.044	1.106	0.984	0.479	0.945	0.046	0.984	0.316	1.018	0.356
**Gender**												
Female vs Male	0.753	0.597	0.320	1.156	0.441	0.136	0.452	0.248	0.597	0.179	0.510	0.138
**AJCC stage**												
I/II vs III/IV	2.457	2.684e-04	366.9	2.427e-05	3.291	3.082e-05	1.942	0.296	2.806	9.227e-10	3.347	2.247e-03
**T stage**												
T1-2 vs T3-4	2.020	1.504e-03	0.007	2.041e-04	2.602	7.525e-04	0.899	0.845	2.272	1.864e-06	0.464	2.048e-02
**N stage**												
N0 vs N1-2	3.943	8.454e-05	41.64	0.011	5.261	3.011e-05	2.439	0.231	4.394	5.446e-09	0.693	0.415
**M stage**												
M0 vs M1	8.301	4.704e04	0.018	4.228e-03	28.38	1.805e-06	3.185	0.291	14.85	2.761e-11	6.087	5.313e-03
**Risk score**												
Low vs High	1.023	9.689e-05	1.047	3.746e-04	10.95	1.042e-06	6.919	1.764e-03	3.308	1.996e-12	3.083	4.494e-06

### Consensus clustering of KIRP patients based on the expression of the three prognostic risk-related m6A RNA methylation regulators

**Figure 5:**
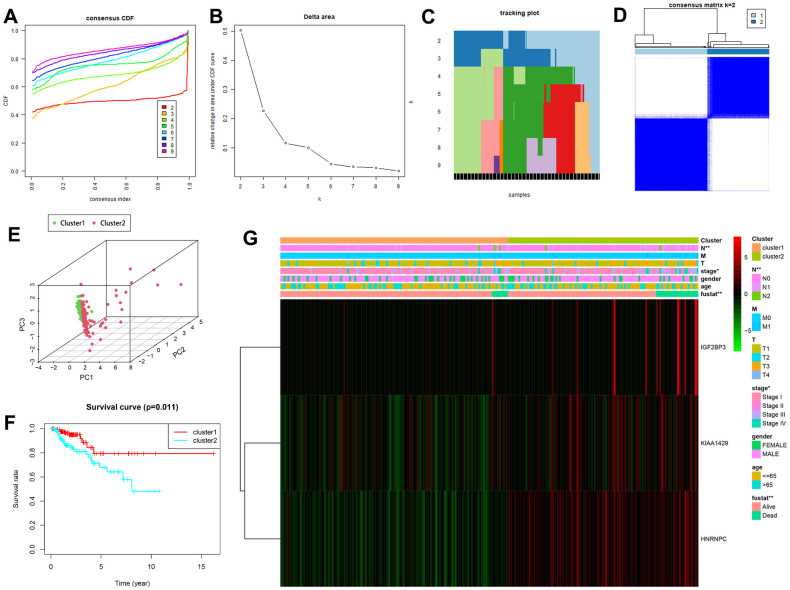
**Consensus clustering analysis shows two clusters of KIRP patients with differential prognosis.** (**A**) Cumulative distribution function (CDF) curves for the consensus score (k = 2 to 9). (**B**) Relative change in area under the CDF curve for k = 2 to 7. (**C**) The tracking plot for k = 2 to 9. (**D**) Consensus clustering matrix for the optimal cluster number, k = 2. (**E**) Principal component analysis shows the gene expression differences between clusters 1 and 2. (**F**) Kaplan-Meier survival curve analysis shows OS rates in cluster 1 and 2 KIRP patients. As shown, OS is significantly shorter for KIRP patients in cluster 2 compared to those in cluster 1. (**G**) The heatmap shows the expression of the three prognostic risk-related m6A methylation regulatory genes in cluster 1 and cluster 2 patients that were stratified according to the clinicopathological parameters, namely, survival status (alive or dead), age (>65 y or <65 y), gender (male or female), AJCC stages (stages I, II, III or IV), T stage (T1-T4), N stage (N0, N1 or N2), and M stage (M0 or M1). As shown, the expression of the three prognostic genes are significantly altered in cluster 1 and cluster 2 patients stratified based on the N stage, AJCC stage and the survival status. * P<0.05; ** P<0.01.

We then performed consensus clustering based on the expression levels of the three m**6**A RNA methylation regulators in the TCGA-KIRP dataset. We chose K = 2 as the most optimal clustering of the TCGA-KIRP patients because the clustering was suboptimal when divided into more than 2 clusters (Figure 5A–5D). Principal component analysis (PCA) also divided the TCGA-KIRP patients into two clusters (clusters 1 and 2) based on their transcriptional profiles (Figure 5E). Subsequently, Kaplan-Meier survival curve analysis showed that the OS was significantly shorter for the KIRP patients in cluster 2 compared to those in cluster 1 (Figure 5F). We then analyzed the correlations between the two clusters and their corresponding clinicopathological features. KIRP patients in clusters 1 and 2 showed significant differences in AJCC stage (*P* < 0.05), N stage (*P* < 0.01) and survival status (*P* < 0.01), but did not show any significant differences in age, gender, T stage and M stage (Figure 5G). Moreover, the expression of m6A RNA methylation modulators was significantly higher in the cluster 2 KIRP patients compared to the cluster 1 KIRP patients.

### Relationship between prognostic risk signature and clinicopathological parameters of KIRP patients

**Figure 6:**
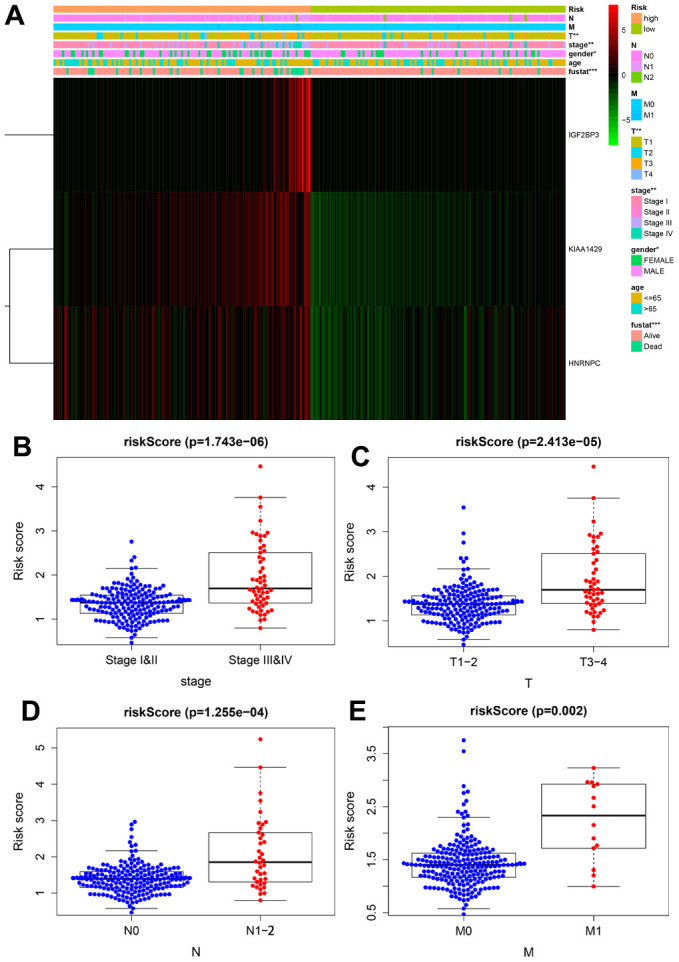
**Correlation analysis between the expression of prognostic risk-related genes and clinicopathological features in high-risk and low-risk KIRP patients.** (**A**) The heatmap shows the expression of the three prognostic signature-related genes in the low- and high-risk group KIRP patients stratified according to the clinicopathological parameters, namely, survival status (alive or dead), age (>65 y or <65 y), gender (male or female), AJCC stages (stages I, II, III or IV), T stage (T1-T4), N stage (N0, N1 or N2), and M stage (M0 or M1). *Chi-square test* evaluated the correlation between the clinicopathological parameters and prognostic risk. *P < 0.05, **P < 0.01 and ***P < 0.001. (**B**–**E**) The distribution of risk scores in high- and low-risk patients stratified according to (**B**) AJCC stage (stages I-II vs. stages III-IV), (**D**) T stage (T1-2 vs. T3-4), (**D**) N stage (N0 vs. N1-2) and (**E**) M stage (M0 vs. M1).

Next, we analyzed the association between the prognostic risk signature and the clinicopathological parameters. The heatmap showed that the expression levels of the three risk-related m6A RNA methylation regulators correlated with the clinicopathological variables in the high- and low-risk groups. We observed significant differences between the high- and low-risk groups in regard to T stage (*P* < 0.01), AJCC stage (*P* < 0.01), gender (*P* < 0.01) and survival status (*P* < 0.001) (Figure 6A). Moreover, advanced-stage tumors significantly associated with the high-risk group, whereas, the early-stage tumors correlated with the low-risk group (Figure 6B–6E).

**Figure 7:**
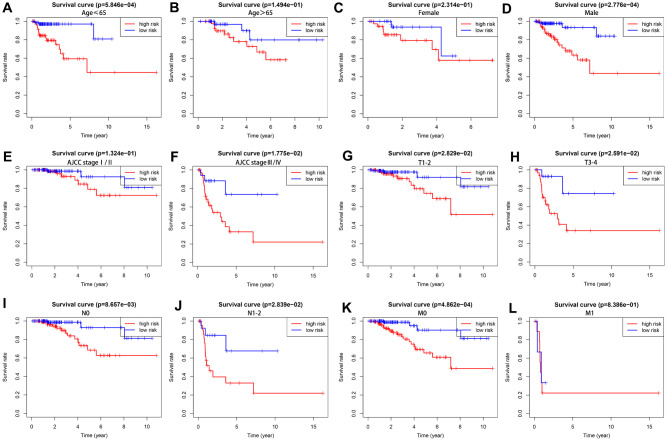
**The overall survival rates in high- and low-risk KIRP patients stratified by clinicopathological parameters.** Kaplan-Meier survival curve analysis shows the OS rates of high-and low-risk KIRP patients stratified by (**A**, **B**) age ≤ 65 and > 65, (**C**, **D**) male and female, (**E**, **F**) AJCC stages I/II and III/IV), (**G**, **H**) T1-2 and T3-4 stages, (**I**, **J**) N0 and N1-2 stages, and (**K**–**L**) M0 and M1 stages.

We further analyzed the prognostic value of our risk score model in different subgroups of KIRP patients that were stratified based on clinicopathological parameters. We observed significantly shorter overall survival rate in the male patients (*P* =2.776e-04) and those with age ≤ 65 (P = 5.846e-04), AJCC stage III/IV (P = 1.775e-02), T1-2 stage (P = 2.829e-02), T3-4 stage (P= 2.591e-02), N0 stage (P = 8.657e-03), N1-2 stage (P=2.839e-02) and M0 stage (P =4.862e-04) in the high-risk group compared to those in the low-risk group (Figure 7). However, OS rates were similar in female patients (P = 2.314e-1), and those with age > 65 (P = 1.494e-1), AJCC stage I/II (P = 1.324e-1), and M1 stage (P = 8.386e-1) in both the high- and low-risk groups.

### Validation of the three prognostic signature-related genes

**Figure 8:**
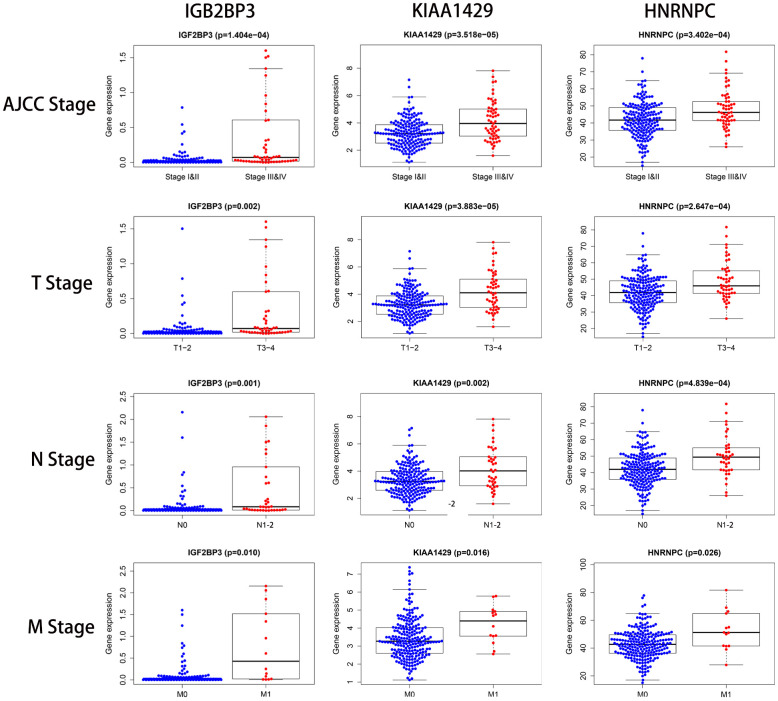
**The relationship between the expression levels of the three prognostic signature-related genes and the clinicopathological features in KIRP patients.** The dot plots show the expression of IGF2BP3, KIAA1429 and HNRNPC genes in KIRP patients belonging to AJCC stages (I&II vs. III&IV), T stages (T1-2 vs. T3-4), N stages (N0 vs. N1-2), and M stages (M0 vs. M1). The p values are shown in parenthesis. As shown, higher expression of the three prognostic-risk related genes correlated with higher AJCC, T, N, and M stages.

Subsequently, we analyzed the correlation between the expression of each of the three prognostic risk signature genes and the clinicopathological features of KIRP patients in the TCGA cohort. We observed differential expression of the 3 risk signature-related genes across various clinicopathological parameters (Table 3). Higher expression of IGF2BP3, KIAA1429 and HNRNPC correlated with higher AJCC, T, N and M stages in the KIRP patients (Figure 8). Moreover, we observed age-dependent differences in the expression of IGF2BP3, and gender-related differences in the expression of KIAA1429 (Table 3).

**Table 3:** Correlation analysis between risk genes from our signature and clinical variables for KIRP.

**Variables**	**Age(≤65, >65) t(*p*)**	**Gender(Female, Male) t(*p*)**	**AJCC Stage(I/II, III/IV) t(*p*)**	**T Stage(T1-2,T3-4) t(*p*)**	**N Stage(N0,N1-2) t(*p*)**	**M Stage(M0, M1) t(*p*)**
IGF2BP3	2.9(0.004)**	1.38(0.171)	-4.086(1.404e-04)***	-3.263(0.002)**	-3.479(0.001)**	-2.998(0.010)*
KIAA1429	0.662(0.509)	3.45(8.505e-04)***	-4.403(3.518e-05)***	-4.443(3.883e-05)***	-3.349(0.002)**	-2.706(0.016)*
HNRNPC	0.353(0.724)	0.458(0.648)	-3.731(3.402e-04)***	-3.848(2.647e-04)***	-3.75(4.839e-04)***	-2.494(0.026)*

t: t value from Student’s t test; p: p-value from Student’s t test.

* p< 0.05; ** p< 0.01; *** p< 0.001.

**Figure 9:**
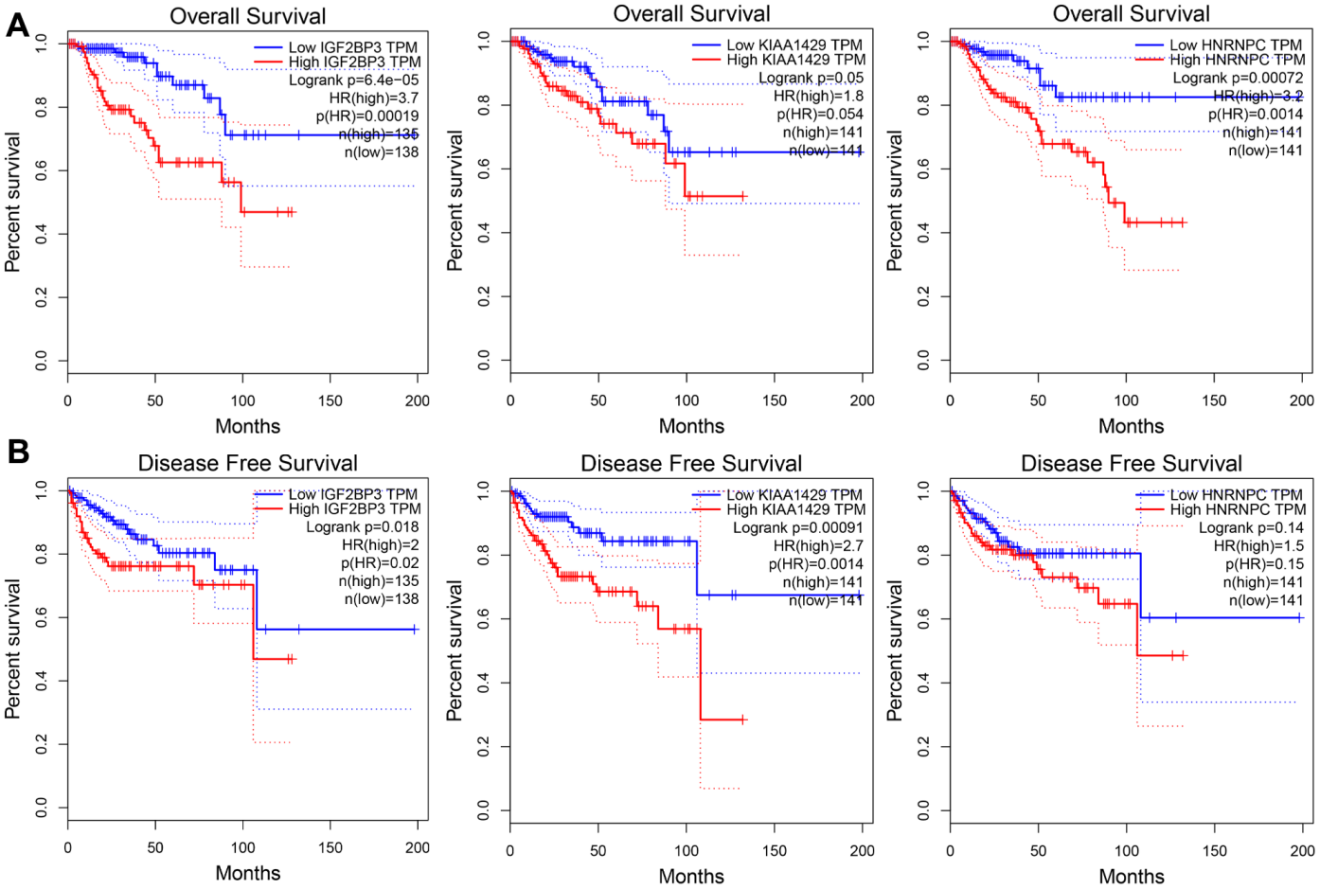
**The overall and disease-free survival rates of KIRP patients from the GEPIA database according to the expression levels of the three prognostic risk signature genes.** Kaplan-Meier survival curves show the (**A**) overall survival (OS) and (**B**) disease-free survival (DFS) rates in KIRP patients from the GEPIA database with high- or low- expression of *IGF2BP3*, *KIAA1429* and *HNRNPC* genes.

We further analyzed the relationship between the risk signature-related genes and the OS and DFS rates of KIRP patients in the GEPIA database. Kaplan-Meier survival curves and log-rank test showed that the OS rate of KIRP patients with higher expression of IGF2BP3 (*P* = 6.4e-05), KIAA1429 (*P* = 0.05) and HNRNPC (*P* = 7.2e-04) was significantly shorter compared to those with lower expression of the three risk signature-related genes (Figure 9A). Moreover, higher expression of IGF2BP3 (*P* = 0.018) and KIAA1429 (*P* = 9.1e-04) correlated with significantly shorter DFS rate (Figure 9B). These data are consistent with our previous findings in the TCGA cohort of KIRP patients. However, we did not find any significant differences in the DFS rates of KIRP patients with differential expression (high or low) of HNRNPC (Figure 9B). Overall, our results demonstrate that the three m6A RNA methylation regulators are potential prognostic biomarkers that can accurately predict survival outcomes of KIRP patients.

## DISCUSSION

KIRP is the second most common renal cancer following clear cell renal cell carcinoma (ccRCC), but, patients with KIRP are often excluded from molecular investigations and randomized clinical trials for kidney cancer because of limited number of cases. Therefore, the molecular mechanisms associated with progression of KIRP are not well understood. Moreover, there is an urgent need to identify effective diagnostic and prognostic biomarkers for early diagnosis and accurate prognosis to improve survival outcomes of KIRP patients. The oncogenic role of several m6A RNA methylation regulators has been reported in several tumors. Hence, in this study, we systematically investigated the prognostic significance of m6A RNA methylation regulators in KIRP.

In the present study, we demonstrated that the abnormal expression of m6A RNA methylation modulators was closely related to tumor progression and survival outcomes in KIRP. Firstly, we demonstrated that 14 out of 20 m6A RNA methylation regulators were differentially expressed in KIRP tissues, including IGF2BP3, IGF2BP1, HNRNPC, YTHDF2, KIAA1429, YTHDF3, METTL14, ZC3H13, ALKBH5, IGF2BP2, RBM15B, YTHDF1, RBMX and HNRNPA2B1. Based on the results of the univariate Cox regression analysis followed by LASSO regression analysis, we constructed a prognostic risk signature with IGF2BP3, KIAA1429 and HNRNPC and classified KIRP patients into high- and low-risk groups based on their risk scores. We demonstrated that the overall survival was shorter for the high-risk patients in the training, testing and the entire TCGA-KIRP cohort compared to the low-risk patients.. We used consensus clustering analysis to categorize the KIRP cohort into two subgroups (cluster1 and cluster 2) according to the expression levels of the three risk-related m6A RNA methylation regulators. Furthermore, univariate and multivariate Cox regression analyses showed that the prognostic risk signature was an independent prognostic factor for KIRP. Overall, our findings demonstrate the predictive value of the three-m6A RNA methylation related gene signature to accurately determine the prognosis of KIRP patients.

Our study demonstrates that IGF2BP3, KIAA1429 and HNRNPC genes are part of a three-gene prognostic prediction signature based on bioinformatics analyses of gene expression profiles of KIRP datasets. IGF2BP3, also known as IMP3, belongs to a conserved IGF2 mRNA-binding protein family. Previous reports have demonstrated that IGF2BP3 is an independent prognostic marker that can be used to identify RCC patients at initial diagnosis who have a high potential to develop metastasis and are candidates for early systemic treatment [23]. Zhou et al. demonstrated that high IGF2BP3 expression correlated with poor survival rates of gastric cancer (GC) patients; moreover, IGF2BP3 knockdown significantly inhibited proliferation and invasion of GC cells [24]. IGF2BP3 has been reported to promote carcinogenesis in colorectal cancer [25], ovarian cancer [26] and pancreatic ductal adenocarcinoma [27]. KIAA1429 is an important methyltransferase that participates in the m6A modification. Qian et al showed that KIAA1429 was associated with *in vitro* and *in vivo* proliferation and metastasis of breast cancer cells [28]. High expression of KIAA1429 was associated with poor prognosis of hepatocellular carcinoma (HCC) patients, whereas, KIAA1429 silencing suppressed *in vitro* and *in vivo* proliferation and metastasis of HCC cells [29]. HNRNPC belongs to a class of proteins that are associated with heterogeneous nuclear RNAs and are involved in the regulation of alternative cleavage and polyadenylation (APA) of mRNAs [30], RNA expression and export [31], post-transcriptional hnRNA stability [32], and cell cycle and apoptosis[33]. Wu et al. showed that repression of HNRNPC inhibits *in vitro* and *in vivo* growth and proliferation of breast cancer cells [34]. These findings are in agreement with our results. We also analyzed the relationship between the three risk-related m6A RNA methylating proteins and the clinicopathological features of KIRP patients and found that increased expression of IGF2BP3, KIAA1429 and HNRNPC significantly correlated with KIRP progression.

To understand the clinical feasibility of the prognosis risk signature for KIRP, we evaluated the association between the risk signature and the clinicopathological parameters. The results showed that the risk signature accurately predicted the survival outcomes of KIRP patients and significantly correlated with their clinicopathological features. Furthermore, we observed two clusters of KIRP patients based on the expression of the three genes in the risk signature. The cluster 1 and 2 proteins showed significantly different OS rate and tumor stages, thereby suggesting that the expression of these three genes correlated with tumor progression. Multivariate Cox regression analysis showed that the three-gene risk signature was an independent prognostic predictor in KIRP patients. Moreover, the risk signature clearly discriminated between early stage or low-risk KIRP patients from advanced stage or high-risk KIRP patients.

Our study has a few limitations. Firstly, the construction and evaluation of our prognostic prediction model was based on the data available in the public databases. Therefore, our results need to be verified by further experimental and clinical investigations. Second, our study failed to identify specific signaling pathways that regulate KIRP growth and progression. Finally, data regarding important clinical variates such as Fuhrman's grade and therapeutic strategy were not available for the KIRP patients in the TCGA database.

In conclusion, we systematically showed that three specific m6A RNA methylation regulators were significantly associated with KIRP progression. We further demonstrated that the prognostic risk signature consisting of IGF2BP3, KIAA1429 and HNRNPC precisely and independently predicted the prognosis of patients with KIRP. Overall, our findings demonstrate that m6A RNA methylation regulatory genes are potential diagnostic and prognostic biomarkers in KIRP.

## MATERIALS AND METHODS

### Patient data

In this study, we obtained RNA-sequencing data of 289 KIRP and 32 normal kidney samples from the TCGA database (https://cancergenome.nih.gov/). We downloaded the corresponding clinical data regarding gender, age, clinicopathological parameters, and survival using the GDC data transfer tool (https://portal.gdc.cancer.gov/). We excluded 14 KIRP patients from the study because their survival time was less than 30 days. We also excluded 38 KIRP patients because of incomplete data. Ethics approval and informed consent were not required for this study because we used publicly available data from TCGA.

### Identification of differentially expressed m6A RNA methylation regulators

We systematically analyzed the expression of 20 m6A-related genes, including seven m6A writers (METTL3, METTL14, WTAP, KIAA1429, RBM15, RBM15B and ZC3H13), two m6A erasers (FTO and ALKBH5), and eleven m6A readers (IGF2BP1, IGF2BP2, IGF2BP3, YTHDF1, YTHDF2, YTHDF3, YTHDC1, YTHDC2, HNRNPC, HNRNPA2B1 and RBMX) in KIRP and non-cancerous kidney samples using the LIMMA package (version 3.30.3; http://bioconductor.org/packages/release/bioc/html/limma.html) from the R software (version 3.6.2; https://cran.rproject.org/bin/windows/base/) and identified differentially expressed genes using P-value < 0.05 as a threshold parameter.

### PPI network and Pearson correlation analysis

We constructed a PPI network between the 14 differentially expressed m6A RNA methylation regulatory genes by downloading the data from the Search Tool for the Retrieval of Interacting Genes (STRING; version 11.0, http://string-db.org) online database [17] and constructing the network with the Cytoscape software (version 3.7.2) between the differentially expressed m6A RNA methylation regulators [35]. Pearson correlation analysis was performed to determine positive or negative association between different m6A RNA methylation regulators based on the Pearson’s correlation coefficient (r) value between −1 and +1.

### Construction of the prognostic risk model

We randomly assigned the 237 KIRP patients to a training cohort (n = 143) and a testing cohort (n = 94). We then performed univariate Cox regression analysis to determine the correlation between differentially expressed m6A RNA methylation regulatory genes and OS of KIRP patients in the training set. We selected the genes showing significant correlation with OS *(P* < 0.05) as prognosis-related genes. Subsequently, we performed the least absolute shrinkage and selection operator (LASSO) Cox regression analysis and identified 3 potential genes to develop the prognostic risk signature. The risk score (RS) of the patients was estimated using the following formula:

$$\text{RiskScore}=\sum_{i=1}^{n}Coef(i)\times x(i)$$

wherein, *Coef*(*i*) and *x*(*i*) represent the estimated regression coefficient and the expression value of each target gene by LASSO analysis, respectively. The risk score formula for the 3-gene prognostic risk signature was as follows: risk score = (0.6715 × expression value of IGF2BP3) + (0.2675 × expression value of KIAA1429) + (0.0109 × expression value of HNRNPC). We then divided the KIRP patients in the training cohort based on their risk scores into high- and low-risk groups by using the median risk score as the cut-off value. We then analyzed the survival parameters of the two groups using the Kaplan-Meier survival curve and two-sided *log-rank test*. We also used receiver operating characteristic (ROC) curves and area under the ROC curve (AUC) values to evaluate the prediction accuracy of our prognostic model. We also performed univariate Cox regression analysis to evaluate the prognostic prediction ability of other clinicopathological factors such as age, gender, AJCC (The American Joint Committee on Cancer) stages, and TNM (Tumor, Node, Metastasis) stages. Multivariate Cox regression analysis was used to assess if the risk score was an independent prognostic factor. The robustness and reliability of the prognostic risk score was then evaluated in the testing cohort (n=94) and the entire TCGA-KIRP cohort (n=237).

### Consensus clustering analysis

We used the Consensus Clusterplus R package to identify different KIRP patient subgroups based on the cumulative distribution function (CDF), delta area, tracking plot and consensus matrix. We evaluated k=2 to 9 potential subgroups for class discovery and clustering validation (50 iterations and 80% resampling rate). Principal component analysis (PCA) was used to assess the signature-related genes expression differences in the two clusters. Kaplan-Meier survival curves and *log-rank* *test* were used to determine the OS rates of KIRP patients in the two clusters. Chi-square test was used to evaluate the differences of clinicopathological characteristics between the two clusters.

### Validation of the clinical utility of the prognostic risk model

Using the Kaplan-Meier (K-M) survival curves, we tested the ability of the prognostic risk signature to predict the survival outcomes of TCGA-KIRP patients stratified by various clinicopathological characteristics, including age (≤ 65 and > 65), gender (female and male), AJCC stage(I/II and III/IV), T stage (T1-2 and T3-4), N stage (N0 and N1-2) and M stage (M0 and M1). The relationship between the expression of the three individual risk signature genes and the clinicopathological characteristics was evaluated using the Student’s *t-test*. We also analyzed the relationship between the risk signature-related genes and the survival parameters (OS and DFS) of KIRP patients in the GEPIA database (http://gepia.cancer-pku.cn/).

### Statistical analysis

All statistical analyses were performed using the R software (version 3.6.2). *Student’s* *t*-test was applied to examine the differences among variables. Pearson’s or *Spearman* correlation analyses were used to determine the association between various parameters. Kaplan-Meier curve analysis with *log-rank test* was used to analyze survival rates between different patient subgroups. *Chi-square tests* were performed to compare categorical variables. Univariate and multivariate Cox regression analyses identify prognostic significance of various clinicopathological characteristics and the prognostic risk score. ROC curve analysis was used to evaluate the efficacy of the prognostic risk model to discriminate between high- and low-risk KIRP patients as well as cluster 1 and cluster 2 KIRP patients based on the AUC values. An AUC value of 1.0 denotes perfect prognostic prediction, whereas an AUC value below 0.5 denotes poor prediction. P < 0.05 was considered statistically significant.
